# A circular bioprocess application of algal-based substrate for *Bacillus subtilis* natto production of γ-PGA

**DOI:** 10.3389/fchem.2023.1158147

**Published:** 2023-04-20

**Authors:** Mattia Parati, Catherine Philip, Barbara Mendrek, David Townrow, Ibrahim Khalil, Fideline Tchuenbou-Magaia, Michele Stanley, Marek Kowalczuk, Grazyna Adamus, Iza Radecka

**Affiliations:** ^1^ Faculty of Science and Engineering, University of Wolverhampton, Wolverhampton, United Kingdom; ^2^ Centre of Polymer and Carbon Materials, Polish Academy of Sciences, Zabrze, Poland; ^3^ Scottish Association for Marine Sciences, Oban, United Kingdom

**Keywords:** ɣ-PGA, circular bioprocesses, industrial up-scaling, macroalgal biomass, GRAS

## Abstract

Poly-γ-glutamic acid (γ-PGA) is a bio-derived water-soluble, edible, hydrating, non-immunogenic polymer. *Bacillus subtilis* natto is a wild-type γ-PGA producer originally isolated from Japanese fermented natto beans whose activity has been shown to be enhanced through ion-specific activation of Extrachromosomal DNA maintenance mechanisms. Being a GRAS γ-PGA producer, this microorganism has attracted great interest in its use within an industrial context. Here we successfully synthesised amorphous, crystalline and semi-crystalline γ-PGA between 11–27 g/L. In line with circular economy principles, scalable macroalgal biomass has been evaluated as substrate for γ-PGA, displaying great potential in both yields and material composition. In this study whole cell, freeze dried seaweed -namely *Laminaria digitata, Saccharina latissima* and *Alaria esculenta-*were pre-treated by means of mechanical methods, sterilised and subsequently inoculated with *B. subtilis* natto. High shear mixing was found to be the most suitable pre-treatment technique. Supplemented *L. digitata* (9.1 g/L), *S. latissima* (10.2 g/L), *A. esculenta* (13 g/L) displayed γ-PGA yields comparable to those of standard GS media (14.4 g/L). Greatest yields of pure γ-PGA were obtained during the month of June for *L. digitata* (Avg. 4.76 g/L) comparable to those obtained with GS media (7.0 g/L). Further, pre-treated *S. latissima* and *L. digitata* complex media enabled for high molar mass (4,500 kDa) γ-PGA biosynthesis at 8.6 and 8.7 g/L respectively. Compared to standard GS media, algal derived γ-PGA displayed significantly higher molar masses. Further studies will be necessary to further evaluate the impact of varying ash contents upon the stereochemical properties and modify the properties of algal media based γ-PGA with the aid of key nutrients; however, the material synthesised to date can directly displace a number of fossil fuel derived chemicals in drug delivery applications, cosmetics, bioremediation, wastewater treatment, flocculation and as cryoprotectants.

## 1 Introduction

Perpetual material circularity can be achieved if nutrient rich industrial by-products are converted into valuable products. Considering the growing desire to replace plastics with biomaterials, our aim is to valorise marine macroalgal waste (MMW) and redeploy this valuable resource as substrate for biosynthetic polymer synthesis. Similarly to other biological resources, seaweed or MMW are disposed of without real valorisation or circularity. Although in line with Waste Framework Directive 2008/98/EC (amended by Directive 2018/851), fresh biomass could be re-deployed towards valuable bioprocesses (Pardilhó et al., 2022). However, seaweed accumulation in coastal regions, spoilage and aesthetic barrier towards tourism prevent these resources from being valorised. Considering the growing concerns towards bioprocess circularity, an array of algal industrial by-products has been explored as potential candidates. For successful valorisation of this biomass, quantitative and qualitative variability are the main barrier towards consistent extraction of bioactives and other valuables (Pardilhó et al., 2022).

Poly-γ-glutamic acid (γ-PGA) is a protein-like compound (pseudo-polypeptide) naturally produced from Generally Regarded As Safe (GRAS) microorganisms, which displays biocompatibility and controllable chemical variability (Ogunleye et al., 2015). From an industrial outlook, chemical versatility, home compostability, water-solubility and non-immunogenicity render this biomaterial particularly suited for a range of applications from bioremediation (Inbaraj et al., 2006; Bai et al., 2022; Deol et al., 2022) to tuneable drug delivery systems (Ye et al., 2006; Khalil et al., 2017; Zhang et al., 2018). As we transition to a biobased economy, these features allow a great reduction in reliance on fossil fuel derived chemicals for several applications (Yang et al., 2021).

**FIGURE 1:**
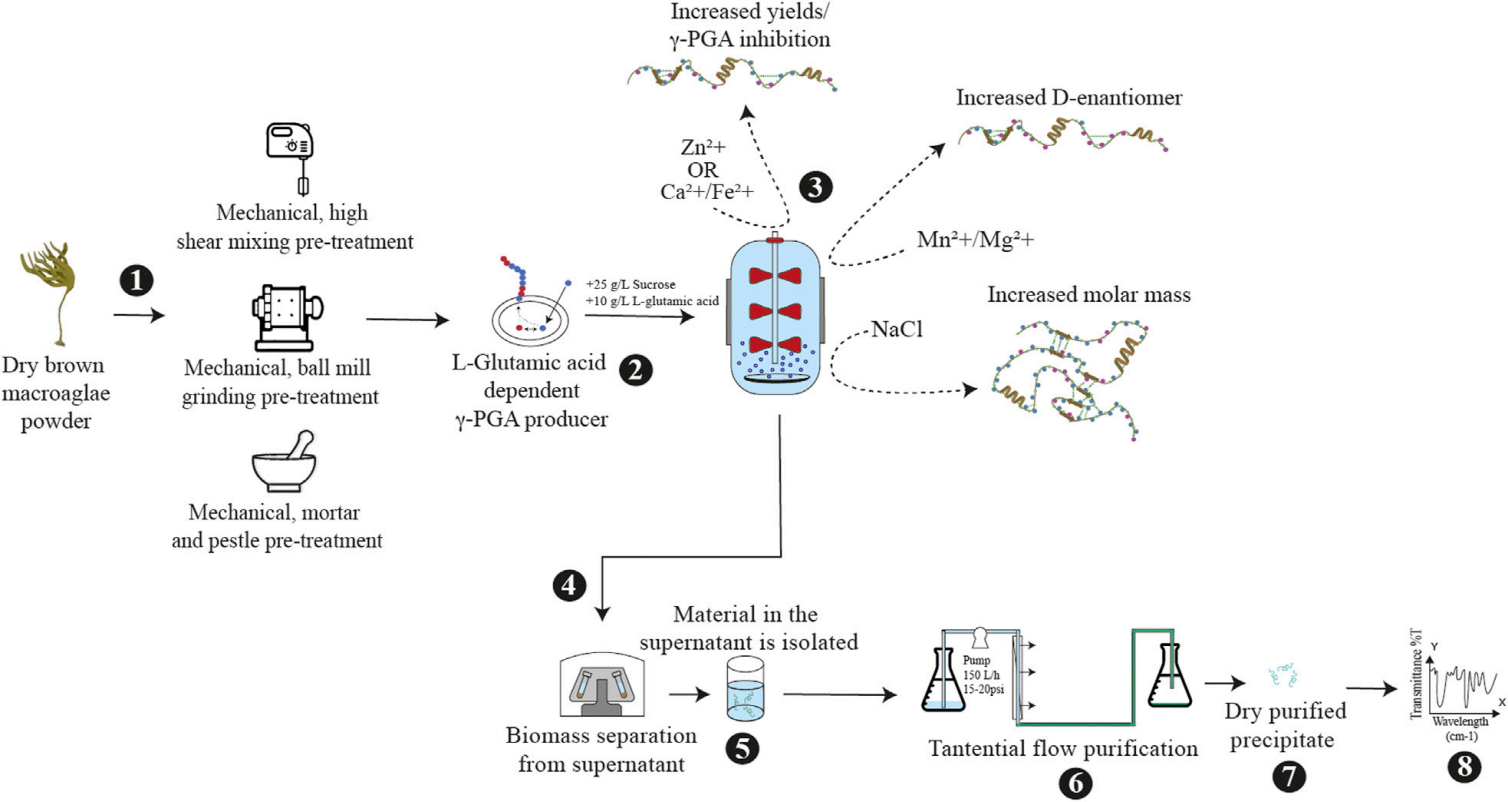
Schematic biosynthesis of cultivated brown macroalgae for γ-PGA biosynthesis. [1] dry macroalgae is subjected to mechanical pre-treatment [2] the pre-treated macroalgae biomass is supplemented with sucrose and glutamic acid; followed by inoculation with a bacterial γ-PGA producer [3] the fermentation media can be supplemented with modulatory compounds, [4] isolation of γ-PGA by centrifugation occurs and [5] γ-PGA is precipitated in ethanol. [6,7] the precipitate is then re-dissolved for further purification [8] followed by freeze drying.

Through enzyme dictated γ-PGA polymerisation, all wild-type producers can tailor the properties of the material in response to environmental queues (Figure 1). To date, numerous *Bacillus* sp. family have been shown to produce large amounts of γ-PGA with varying molar masses and stereochemical properties (Parati et al., 2022). As introduced by Ashiuchi et al. (1999) and later described by Yamashiro et al. (2011) the polyglutamate synthase (pgs) intermembrane enzymatic complex is responsible for the polymerisation of D/L-glutamic acid racemic mixtures. The peculiarity of this biosynthetic mechanism is that the enzymatic complex is directly responsible for gamma-peptide bond formation. Numerous research groups have attempted to elucidate the effect of different ions on the activity of the pgs complex. Fine-tuning chemico-phyisical properties of the material would unlock the potential for application-specific industrial biosynthesis. Similarly to other enzymes, the presence of different ions can drastically affect the activity of one or more biocatalytic components within the pgs complex, which result in a significant variation in γ-PGA properties (Parati et al., 2022). For instance, the presence of manganese–Mn^2+^ (Birrer et al., 1994; Kedia et al., 2010), zinc–Zn^2+^ (Yamashiro et al., 2010), sodium–Na^+^ (Birrer et al., 1994; Kedia et al., 2010) (Ogawa et al., 1997; Ashiuchi et al., 2006; Ashiuchi and Shimizu, 2015) calcium–Ca^2+^ (Yamashiro et al., 2010), magnesium–Mg^2+^ (Ashiuchi and Shimizu, 2015) and iron–Fe^2+^ (Yamashiro et al., 2010) ions have all been shown to affect the yields and/or chemical characteristics of the biomaterial (Ashiuchi et al., 2001; Ashiuchi et al., 2007). Understanding the factors and elements which determine polymeric properties is vital to reproducibly synthesise, application-specific materials (Parati et al., 2022). The nuances of such biochemical mechanisms have been further discussed by Ashiuchi et al. (1999) and Yamashiro et al. (2011).

Currently, the biosynthesis of γ-PGA is predominantly achieved by cultivation of bacteria in defined media, often too expensive for low value commodity applications (Zhang et al., 2019; Parati et al., 2022). With an outlook on material up-scaling and industrial commercialisation, significantly cheaper, complex media have been assessed and shown to be suitable for γ-PGA biosynthesis. For instance, Nair et al. (2021) employed rotten tomatoes for the biosynthesis of γ-PGA, Kim et al. (2019) synthesized γ-PGA from the macroalgae *Ulva* sp., Parati et al. (2022) synthetised γ-PGA from supplemented micro and macroalgae, Bajaj et al. (2008) employed soybean meal for the biosynthesis of γ-PGA. The use of other complex waste media, including brewery waste for γ-PGA biosynthesis has been further discussed by Parati et al. (2022b).

**FIGURE 2:**
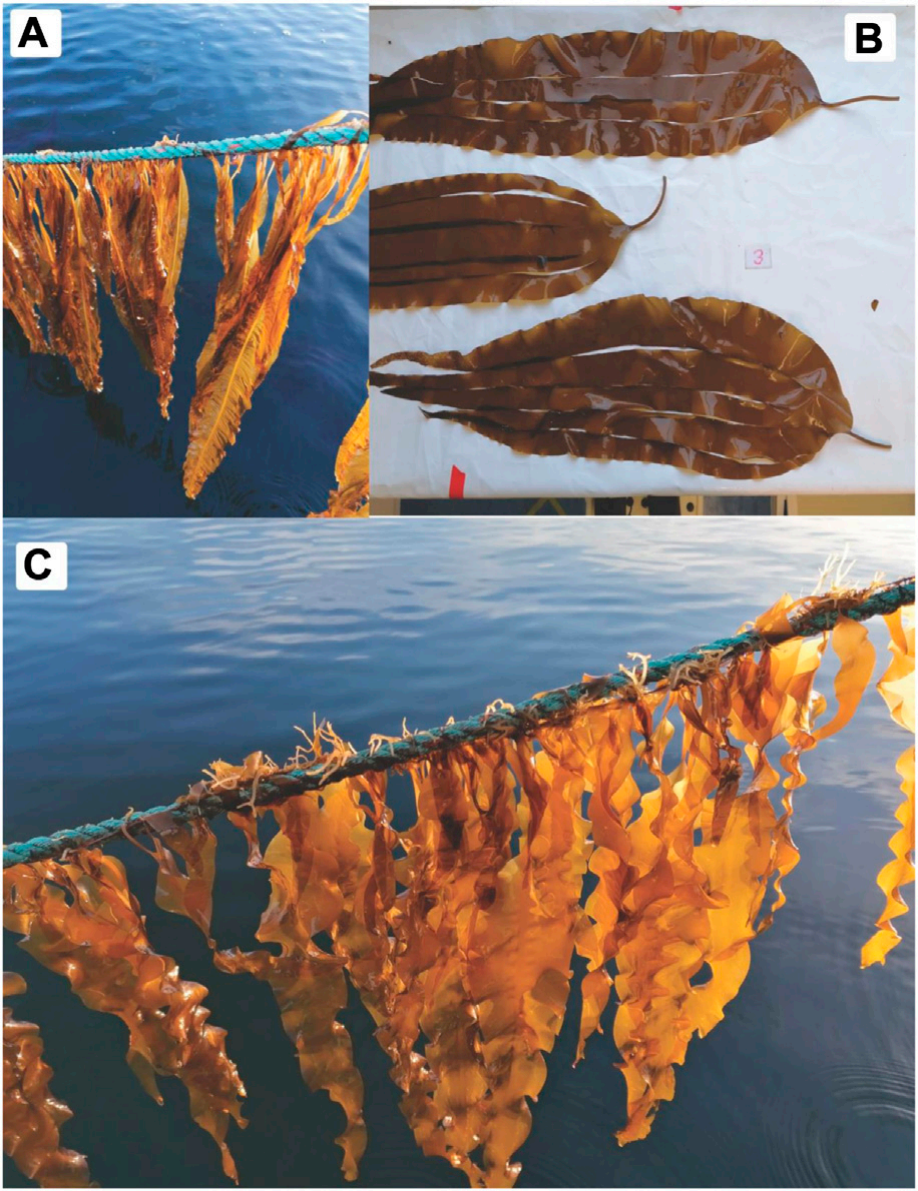
Depiction of **(A)** *Alaria esculenta,* **(B)** *Laminaria digitata*, and **(C)** *Saccharina latissima* during or after cultivation on standard rope systems in Oban, United Kingdom. Pictures provided by the Scottish Association for Marine Science (SAMS).

Some of the biggest challenges faced when employing complex media include seasonal availability of the substrate, its pre-treatment and the variation in its composition. All of which can affect the supply and quality of resulting added value products (Parati et al., 2022; Pardilhó et al., 2022). Here we assessed the impact of pre-treatment type, macroalgal species and time-year collection of brown seaweed cultivated under controlled conditions by the Scottish Association for Marine Science, Oban, Scotland. Seaweed has been known to be a fantastic source of carbohydrates, proteins, antioxidants and fibrous material; and with increasing interest in their components, numerous nations are abandoning wild harvests and stimulating local cultivation of these fast growing, highly carbon sequestering organisms. Endemic to United Kingdom coast, *Laminaria digitata*, *Saccharina latissima* and *Alaria esculenta* are three macroalgal species which grow throughout the year (Figure 2) and, although are all part of the brown seaweed family, all display great variation in carbon, antioxidant, proteinaceous material and ash content/composition (Schiener et al., 2014; Pardilhó et al., 2022). To this end we assessed the effect of different algal species on the yields and chemical composition of γ-PGA from *Bacillus subtilis* natto. The impact of polymeric biochemical variation occurring during the cultivation period (April to August) on *B. subtilis* natto γ-PGA biosynthesis was also evaluated.

Here we report the biosynthesis of γ-PGA in standard media as well as the potential of macroalgae to provide a cost effective and scalable solution towards the biosynthesis of γ-PGA. Additionally we have investigated the effect of standard and algal based cultivation media on yields and polymer chemistry.

## 2 Materials and methods

### 2.1 Biosynthesis of ɣ-PGA

#### 2.1.1 Microorganism

*Bacillus subtilis* natto (ATCC15245) obtained from the National Collection of Industrial and Marine Bacteria (NCIMB) were freeze-dried and kept at −20°C. Before use, cultures were resuscitated and grown on Tryptone Soya Agar (TSA) (Lab M, Heywood, UK) overnight at 37°C. Highly mucoid colonies were selected and grown aerobically in shake flasks containing 100 mL of Tryptone Soya Broth (TSB) medium (Lab M, Heywood, UK) at 37°C for 24 h.

#### 2.1.2 Media

##### 2.1.2.1 Standard media.

Standard GS Medium (50 g/L NaCl) was composed of 20 g/L L-glutamate purchased from Fischer Chemicals Ltd. (Loughborough, UK), 50 g/L sucrose, 2.7 g/L KH_2_PO_4_, 4.2 g/L Na_2_HPO_4_, 50 g/L NaCl, 5 g/L MgSO_4_ ·7H_2_O, and 1 mL/L of Murashige-Skoog vitamin solution, all purchased from Sigma-Aldrich (Irvine, UK). The pH of this medium was adjusted to 6.8 using NaOH purchased from Fischer Chemicals Ltd. (Loughborough, UK).

Modified GS Medium (25 g/L NaCl) was composed of 20 g/L L-glutamate purchased from Fischer Chemicals Ltd. (Loughborough, UK), 50 g/L sucrose, 2.7 g/L KH2PO4, 4.2 g/L Na2HPO4, 25 g/L NaCl 5 g/L MgSO4 ·7H2O, and 1 mL/L of Murashige-Skoog vitamin solution, purchased from Sigma-Aldrich (Irvine, UK). The pH of this medium was adjusted to 6.8 using NaOH purchased from Fischer Chemicals Ltd. (Loughborough, UK). Modified media does not contain 2.78 mMol (0.42 g/L) Manganese Sulphate unless otherwise stated.

Modified GS Medium (0 g/L NaCl) was composed of 20 g/L L-glutamate purchased from Fischer Chemicals Ltd. (Loughborough, UK), 50 g/L sucrose, 2.7 g/L KH_2_PO_4_, 4.2 g/L Na_2_HPO_4_, 5 g/L MgSO_4_ ·7H_2_O, and 1 mL/L of Murashige-Skoog vitamin solution, purchased from Sigma-Aldrich (Irvine, UK). The pH of this medium was adjusted to 6.8 using NaOH purchased from Fischer Chemicals Ltd. (Loughborough, UK). Modified media does not contain 2.78 mMol (0.42 g/L) Manganese Sulphate unless otherwise stated.

##### 2.1.2.2 Complex media.

All macroalgal complex media were composed of 40 g/L seaweed (origin specified within Table 1) and supplemented with 10 g/L L-glutamate (Fischer Chemicals Ltd., Loughborough, UK) and 25 g/L Sucrose (Sigma-Aldrich, Irvine, UK). The pH of all media was adjusted to 6.8 using NaOH purchased from Fischer Chemicals Ltd. (Loughborough, UK).

**TABLE 1:** Summary of complex algae media, their pre-treatment and figures which display their average yields in γ-PGA.

Media name	Algae component(s)	Description
Complex Medium 1	40 g/L Commercial *Laminaria digitata* flakes undergone pre-treatment investigations (Cornish Seaweed Ltd., United Kingdom)	Commercial sample subjected to mechanical mortar and pestle or high shear mixing or ball mill grinding pre-treatment
Complex Medium 2	40 g/L cultivated *Laminaria digitata* pre-treated with high shear mixing (SAMS, Oban, United Kingdom)	Cultivated macroalgae collected and analysed for γ-PGA production at six timepoints during the year between 07.04.2021 and 01.07.2021
Complex Medium 3	40 g/L of cultivated *Laminaria digitata* or *Alaria esculenta* or *Saccharina latissima* pre-treated with high shear mixing (SAMS, Oban, United Kingdom)	γ-PGA yields from each algal group cultivated 07.04.2021 and 01.07.2021 were averaged to assess variation in yields during isolation at different yearly timepoints

All complex media were prepared using deionised water and sterilised by autoclaving at 115°C for 15 min. The sucrose solution was filter sterilised separately (0.22 µm, Fischer Chemicals Ltd., Loughborough, UK) and added separately to the medium.

#### 2.1.3 Cultivation parameters

Batch cultures were carried out in 250 mL shake flasks. The cultures were inoculated by thawing the frozen cells in a 37 °C bath (Grant Instruments, Fischer Chemicals Ltd., Loughborough, UK) and 5% (v/v) of inoculum was added to the cultivation media. Growth temperature was kept at 37°C, agitation was set 150 rpm for the 96 h of cultivation period.

3Medium scale batch cultures were carried out in a 7-L fermenter with a 4-L working volume (Electrolab, Tewkesbury, UK). The media were inoculated at a 5% (v/v) with a 24 h starter culture. The cultivation temperature was kept at 37°C, and the pH of the culture was maintained at 6.8 with the aid of 3 M NaOH (Fischer Chemicals Ltd. Loughborough, UK) or 3 M HCl (Honeywell, Reading, UK). The stirring speed and airflow were set to 150 rpm and 2.0 L/min respectively at the start of fermentation. Provided that the viscosity of the fermentation media increased significantly during the cultivation of *Bacillus subtilis* natto, both stirring speed and airflow were adjusted to keep the supply of oxygen above 40%. To this end, the agitation speed was increased to 250 rpm and air flow rate was increased to 3 L/min.

Colony forming units (CFU/mL) was monitored periodically by aseptically removing 0.5 mL aliquots and diluting them sequentially in one-quarter strength Ringer solutions (Lab M, Heywood, UK). For each sample, 0.5 mL was serially diluted in 10-fold steps to 10^–7^. Ringer solution was prepared by dissolving 1 tablet in 500 mL of deionised water in a 1-L flask. Cell viability was determined by serial dilutions onto Petri dishes containing nutrient agar. Each dilution was plated following the Miles and Misra method (Miles et al., 1938) and employing 20 µL of each dilution. Colonies were counted after overnight incubation at 37 °C and organismal concentration was expressed as Log_10_ [CFU/mL].

### 2.2 Isolation and purification of γ-PGA

For material isolation, the culture broth was withdrawn from the fermentation vessel and centrifuged at 17,000 × *g* for 30 min to remove cells, by employing a ERMLE Z 300K centrifuge (Wehingen, Germany). The supernatant was poured into 3 volumes of cold ethanol and left overnight at 5°C to precipitate in static conditions. The resultant precipitate was collected by filtration over a 0.22 µm paper filter (Fischer Chemicals Ltd., Loughborough, UK). The precipitate was subsequently lyophilised (Alpha one to four LSC plus Christ Freeze Dryer, SciQuip Ltd. Bomere Heath, UK) with a 36 h run. The white/green/brown, dry powder was stored in a desiccator at room temperature until further use.

For further purification, the obtained dry powder was re-dissolved into deionised water and dialysed against deionised water within a cross flow system purchased from Repligen, US with a 20 cm MidiKros column of 30 KDa cut off from Repligen. The purified solution was re-precipitated with 3 volumes of cold ethanol and left at 5 °C overnight. After precipitation the polymer was collected and lyophilised.

### 2.3 Statistical analysis

All cultivation parameters and yield presented were undertaken in triplicates. Results were statistically analysed by means of standard deviation, standard error and two-way ANOVA using Graphpad prism (unless otherwise specified). All results were statistically analysed using Microsoft Excel and SPSS 25.

### 2.4 Characterisation of γ-PGA

The purified polymer isolated form was identified by fourier transform infrared spectroscopy (FT-IR) with Nicolet 380 FT-IR (Thermo Fisher Scientific Inc., Wilmington, DE, USA) with 32 scans and 4 cm^−1^ resolution. The measurements were over 100 scans and wave number range of 400–4,000 cm^−1^.

The average molar mass and molar mass distributions of the polymers were determined by gel permeation chromatography (GPC) with a differential refractive index detector (Δn-2010 RI WGE Dr. Bures, Berlin, Germany) and a multiangle laser light scattering detector (DAWN EOS, Wyatt Technologies, Santa Barbara, CA, USA). The following set of columns was used: guard PSS SUPREMA 10 μm and PSS SUPREMA analytical Linear XL 10 μm + PSS SUPREMA Ultrahigh 10 μm (Polymer Standards Service, Mainz, Germany). Buffer (0.15M NaNO_3_, 0.01M EDTA, 0.02% NaN_3_ and pH = 6 adjusted with NaOH) was used as solvent with nominal flow rate of 0.5 mL/min. Measurements were done at 40°C. ASTRA 4 software (Wyatt Technologies, Santa Barbara, CA, USA) was used to evaluate the results. All samples were filtered through the 0.45 µm PES syringe filters (Graphic Controls, DIA-Nielsen, Düren, Germany) before measurements. The refractive index increment of commercial γ-PGA was estimated in independent measurement in buffer using a differential refractive index detector. (SEC-3010 dn/dc WGE Dr. Bures, Berlin, Germany) and was equal 0.142 mL/g.

To attain high quality diffraction data for γ-PGA samples, a Panalytical Empyrean (Malvern, UK) X-ray diffractometer with a Cu anode X-Ray tube was employed. For sample analysis, approximately 200 mg of pure γ-PGA powder was homogeneously flattened within the holder with the aid of a glass slide. Analysis conditions employed were: Scan Axis: Gonio, Start Position [°2Th.]: 5.0090, End Position [°2Th.]: 99.9870, Step Size [°2Th.]: 0.0130, Scan Step Time [s]: 8.6700, Irradiated Length [mm]: 15.00, Specimen Length [mm]: 10.00, Measurement Temperature [°C] 25.00, Generator Settings: 40 mA, 40 kV, Goniometer Radius [mm]: 240.00, Dist. Focus-Diverge. Slit [mm]: 100.00.

Size analysis of the particles was undertaken by using laser diffraction on a Malvern Mastersizer 3000 (Malvern, UK) fitted with its Hydro-EV system. Samples were analysed by aqueous suspension at an obscuration level of 15%. Particles were predicted to be irregular with spherical-like shape. The refractive index of the material was set to 1.53 with an absorption index of 0.1 and a density value of 0.3 g/cm^3^. Five measurements per each sample were undertaken, the data was then processed by means of the Mastersizer 3000 software v3.88 with which the average was calculated.

## 3 Results

### 3.1 Growth of *Bacillus subtilis* natto on standard media

**FIGURE 3:**
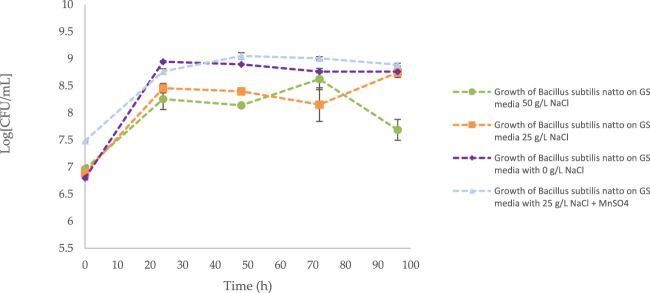
Growth curve of *Bacillus subtilis* natto cultivated on standard media. Error bars indicate standard error (n = 6).

Growth of *B. subtilis* natto on standard media has been summarised within Figure 3. Presented growth curves from Figure 3 suggest that high concentrations of NaCl (50 g/L) result in decreased cell viability between t = 72 and t = 96 h (*p* < 0.0001). Differently, our data suggests that there is not a significant difference in growth with defined media between 0 g/L and 25 g/L of NaCl (*p* > 0.05). Further, the addition of 2.78 mMol MnSO_4_ appears to have similar cellular proliferation and viability compared to 0 g/L and 25 g/L NaCl concentrations.

**FIGURE 4:**
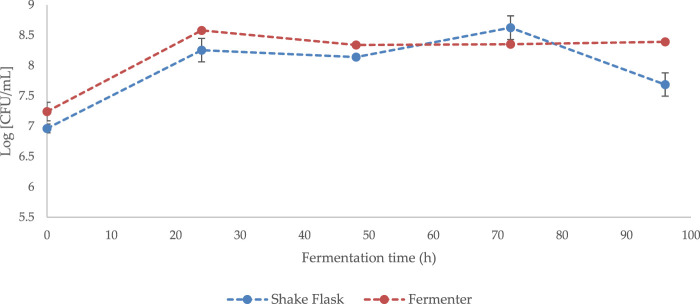
Growth curve of *Bacillus subtilis* natto cultivated on standard GS media (50 g/L NaCl) in shake flaks (working volume 250 mL) or in benchtop fermenter (working volume 4,000 mL). Error bars indicate standard error (n = 3 fermenter, n = 6 shake flask).

During the up-scale phase, an increase in cultivation volume from 250 mL (shake flask) to 4,000 mL (bench top fermenter) does not appear to have a significant effect on *Bacillus subtilis* natto cell growth or viability (*p* > 0.05) (Figure 4).

### 3.2 Particle size of complex algal media

**FIGURE 5:**
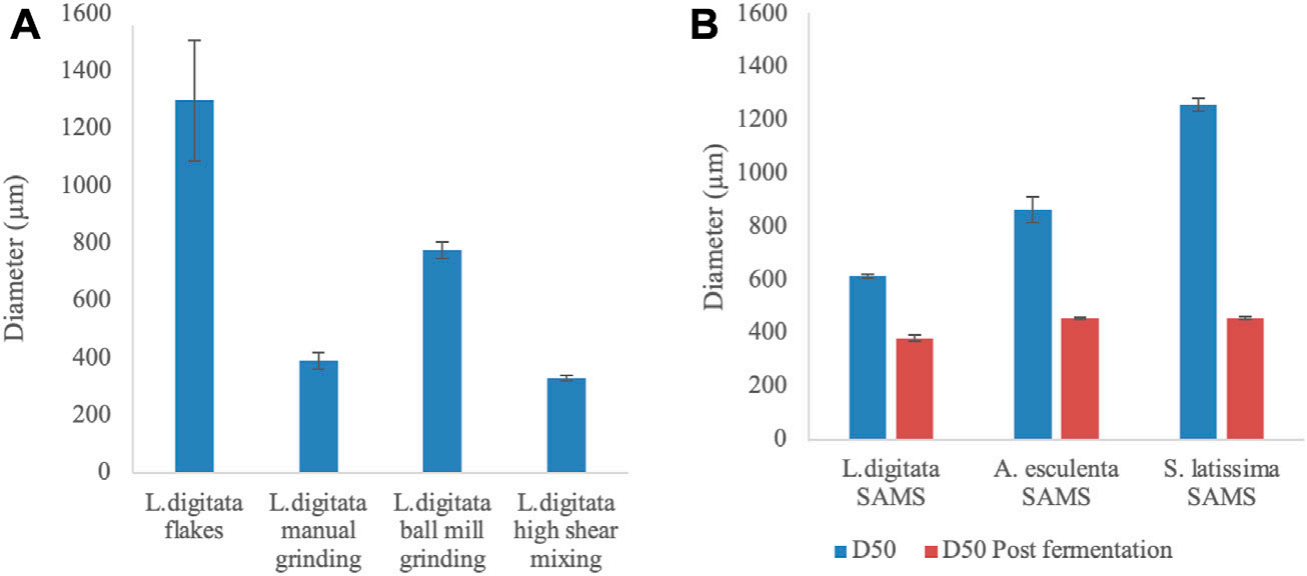
Average diameter [Dx (50)] of algal samples undergone different mechanical pre-treatments. **(A)** Mechanical processing of commercially available *Laminaria digitata* flakes **(B)** Variation in diameter of particles provided by Scottish Association for Marine Science (SAMS - Oban, UK) before and after production of γ-PGA. All samples are the average of five replicates processed by the machine.

**FIGURE 6:**
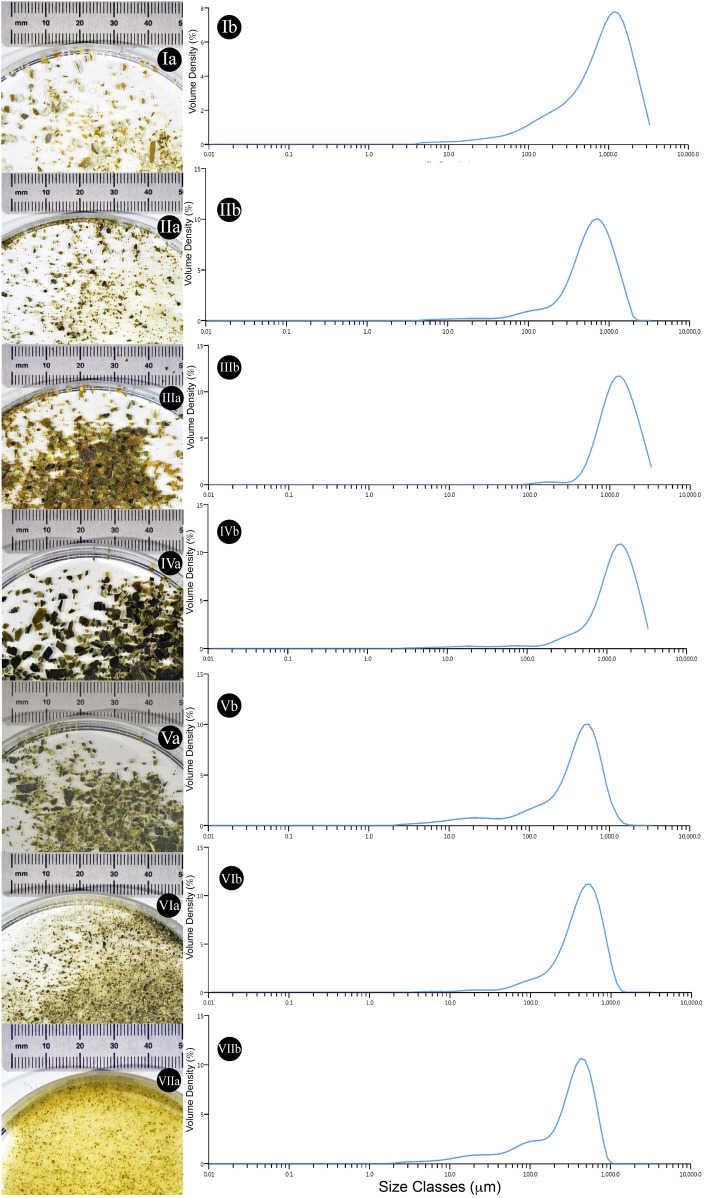
Qualitative and quantitative particle size evaluation from different algal samples. **(Ia)** Ground *Alaria esculenta* starting material provided by SAMS (Oban, UK) **(Ib)** Average particle size dispersity of the starting material (n = 3). **(IIa)** Ground *Laminaria digitata* starting material provided by SAMS (Oban, UK) **(IIb)** Average particle size dispersity of the starting material (n = 3). **(IIIa)** Ground *Saccharina latissima* starting material provided by SAMS (Oban, UK) **(IIIb)** Average particle size dispersity of the starting material (n = 3) **(IVa)** Commercial *Laminaria digitata* flakes **(IVb)** Average particle size dispersity of the starting material (n = 3). **(Va)** Commercial *Laminaria digitata* flakes undergone mortar and pestle pre-treatment **(Vb)** Average particle size dispersity of the starting material (n = 3). **(VIa)** Commercial *Laminaria digitata* flakes undergone ball mill grinding pre-treatment **(VIb)** Average particle size dispersity of the starting material (n = 3). **(VIIa)** Commercial *Laminaria digitata* flakes undergone water based high shear mixing pre-treatment **(VIIb)** Average particle size dispersity of the starting material (n = 3).

The initial, and post mechanical treatment, particle sizes of algal fractions were analysed to evaluate potential variation in nutrient availability during the cultivation. The diameter of different particles has been reported within Figures 5, 6.

When different mechanical pre-treatment methods were employed, a significant variation in particle size was observed (*p* < 0.0001) (Figure 5A). Across pre-treatment methods, it appears that no significant variation in particle size was observed between manual grinding and high shear mixing (*p* > 0.99). Figure 5A also suggests that the particle size obtained with manual grinding is lower compared to ball mill grinding; however, these results appear to differ compared to visual observations (see Figures 6V–VI).

In addition, algal samples provided by the Scottish Association for Marine Science (SAMS–Oban, UK) were analysed for the initial particle size and further subjected to analysis after γ-PGA cultivation. Figure 5B suggests that there is significant variation in initial algal particle size across the three species (*p* < 0.0001) with analogous results post high shear mixing and γ-PGA production (*p* < 0.0001).

### 3.3 Growth of *Bacillus subtilis* natto on complex algal media

**FIGURE 7:**
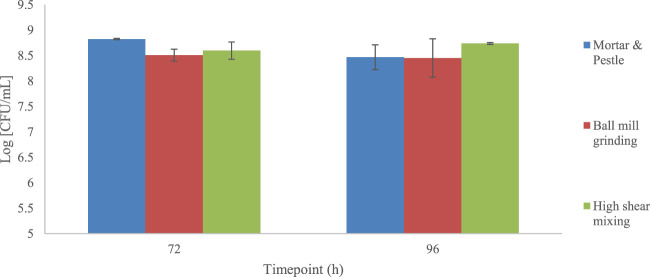
Concentration of *B. subtilis* cells cultivated on commercial *Laminaria digitata* flakes undergone different mechanical pre-treatment methods and substrate composition. All samples were supplemented with 25 g/L sucrose and 10 g/L l-glutamic acid. Error bars indicate standard error (n = 6).

During substrate optimisation studies, different seaweed pre-treatments were assessed. Three mechanical pre-treatment techniques were investigated: ball mill grinding, mortar and pestle and high shear mixing. The results of this investigation have been summarised within Figure 7. The growth of *B. subtilis* at different time points was significantly different (*p* < 0.01) across the three pre-treatment techniques. Further, the growth of *Bacillus subtilis* natto on a commercial *Laminaria digitata* flake media (no pre-treatment) with addition of 7 g/L L-glutamic acid only was attempted to benchmark this algal specie to results reported by Kim et al. (2019). Only one of the three replicates displayed growth across all time intervals with a final concentration of 6.43 CFU/mL; suggesting that the media was inadequate for cell growth (data not shown). This dataset allowed us to understand that, although a potentially good source of nutrients, both pre-treatment and supplementation of algal flakes was preferable to achieve high concentrations of *B. subtilis* natto.

### 3.4 Identification of γ-PGA

**FIGURE 8:**
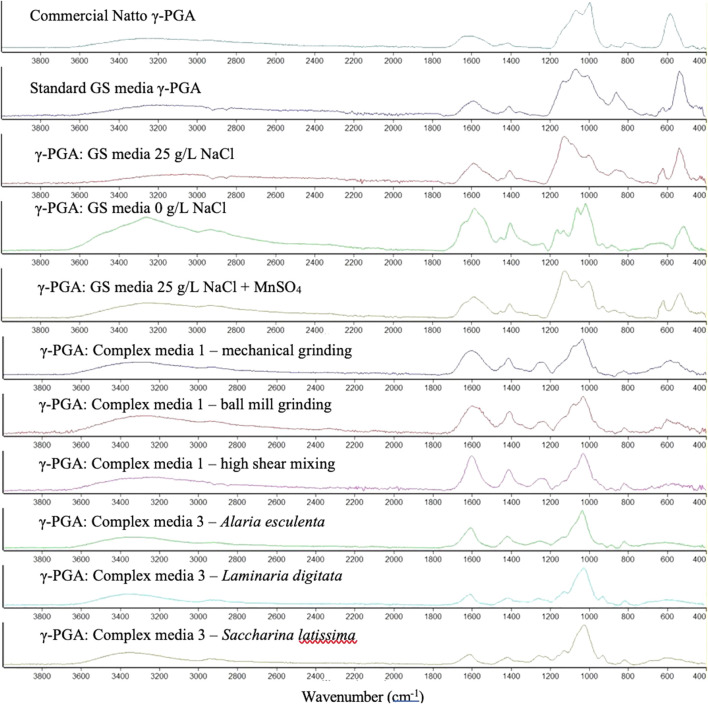
Absorbance Fourier Transform Infra-Red spectra of γ-PGA obtained from different standard and complex waste substrates after tangential flow filtration. The peaks within the figure represent different functional groups of γ-PGA: C=O stretch (1739 cm^−1^), the Amide I N-H bend (1643 cm^−1^), the Amide II stretch (1585 cm^−1^), the C=O symmetric stretch of γ-PGA in its sodium (1402 cm^−1^) or calcium (1412 cm^−1^) isoform, the C-N stretch (1162 cm^−1^), the N-H bending (689 cm^−1^), the O-H stretch (3449 cm^−1^).

The chemical composition of γ-PGA was investigated with the aid of FT-IR analytical technique. Figure 8 suggests that γ-PGA is in fact produced. However, surprisingly it does not appear that complex algal waste media yields significant variation in purity compared to commercial sample of γ-PGA after tangential flow filtration.

### 3.5 ƴ-PGA yields from standard media

**FIGURE 9:**
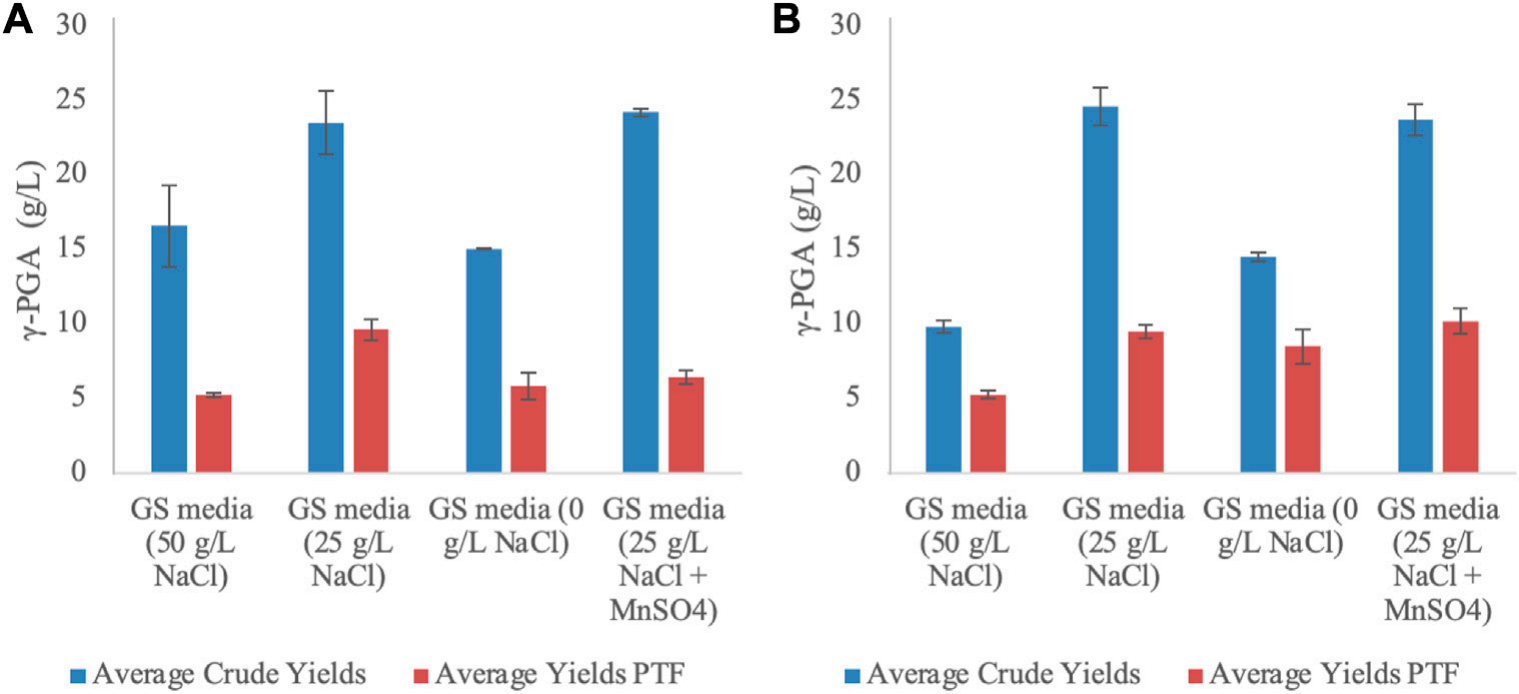
Average yields of γ-PGA (crude and PTF) obtained from standard cultivation media at varying NaCl concentrations in 4L fermenter **(A)** and 250 mL shake flask **(B)**. Error bars indicate standard deviation (n = 3).

Following collection of the fermentation media from the vessel, the media were centrifuged and subsequently γ-PGA was isolated by means of solvent precipitation (Figure 1). The crude precipitate is known to contain an array of low molar mass impurities with similar chemical composition to γ-PGA. Additional purification of γ-PGA is achieved with tangential flow filtration, wherein peptides and other small impurities (i.e., amino acid) impurities pass through 30,000 Da pores with γ-PGA being maintained within the retentate. Overall, both shake flasks and 4L fermenters suggested significant variation in both raw yields (*p* < 0.0001 and *p* = 0.0016 respectively one way ANOVA) and Post Tangential Flow (PTF) yields (*p* = 0.0004 and *p* = 0.0018 respectively one way ANOVA). Figure 8 suggests that for standard GS media 4L fermentation, the highest yields of γ-PGA are obtained with MnSO_4_ supplemented 25 g/L NaCl media. This is the opposite in shake flask experiments (see Figure 9). However, there is no significant difference in yields between GS media 25 g/L NaCl and 25 g/L NaCl + MnSO_4_ in either shake flasks (*p* = 0.59) or 4L fermenters (*p* = 0.71) (Figure 8). Similarly, there is no significant difference in yields between 0 and 50 g/L NaCl media in shake flasks (*p* = 0.006) or in 4L fermenters (*p* = 0.99) (Figure 9). Differently, there is a significant variation in yields between cultivation with 25 g/L NaCl compared to 0 or 50 g/L NaCl (*p* < 0.0025). Overall, there was no significant variation in raw yields obtained between cultivation in shake flask or 4L fermenter (*p* = 0.27).

When the material was further purified, yields obtained from shake flasks were significantly different (higher) compared to those obtained from 4L fermenter (*p* = 0.0164). Overall, the highest yields of pure γ-PGA were obtained in 0 g/L NaCl media (61% (w/w)) for shake flasks, whereas they were obtained in 0 and 25 g/L NaCl media for 4L fermenters (39 and 41 (w/w%)). Overall higher yields of pure material were obtained from shake flasks compared of 4L fermenters. During further scale up, cultivation parameters will need to be further optimised.

### 3.6 ƴ-PGA yields from different mechanical pre-treatments

**FIGURE 10:**
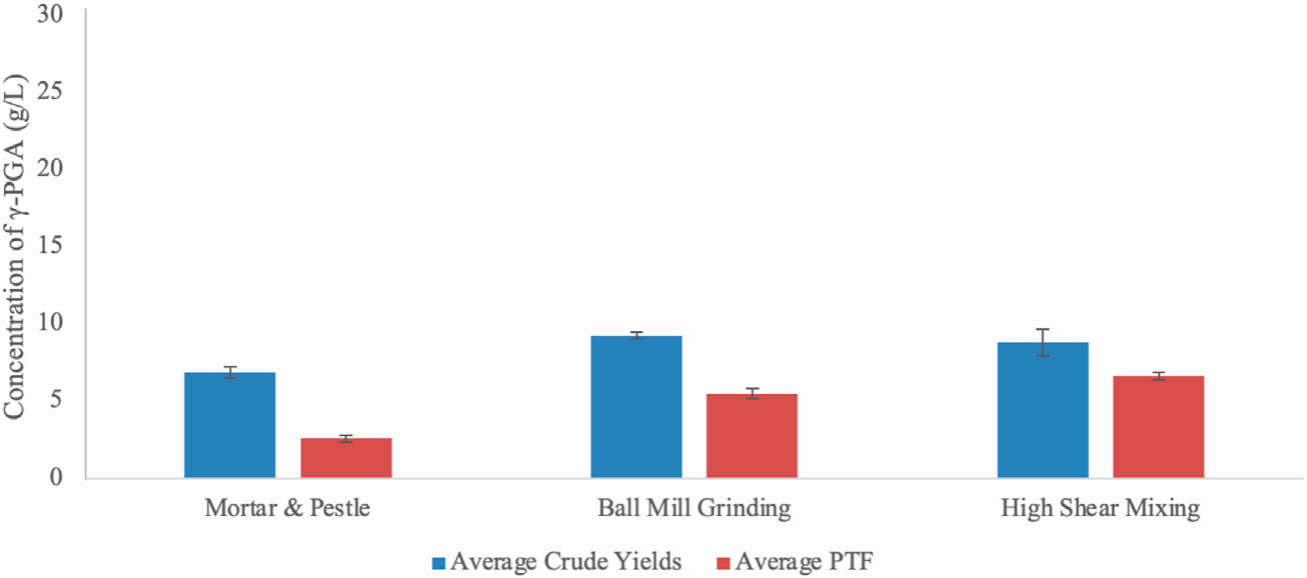
Average yields of γ-PGA (raw and PTF) obtained from *Laminaria digitata* commercial flakes undergone different pre-treatment techniques. Error bars indicate standard deviation (n = 3 shake flask).

When it comes to industrial scalability, employing refined sugars and amino acids becomes economically and environmentally unsustainable (Dunn et al., 2017). To this end, we assessed the benefit of employing brown algal biomass as a substrate for the biosynthesis of γ-PGA from *B. subtilis* natto. Availability of micro and macronutrients contained within algal cells can only be employed into the biosynthesis of γ-PGA if released into the growth media. The results are presented within Figure 10.

Data summarised within Figure 10 suggests that there is a significant difference in γ-PGA yields between raw and post tangential flow treatment samples (*p* = 0.0001). Further, data suggests that pre-treatment significantly influences raw and PTF γ-PGA yields (*p* = 0.0039 and *p* < 0.0001, respectively). As expected, the percentage of γ-PGA following tangential flow treatment increased between pre-treatment methods from 37.5 (w/w%) (manual) to 59.6 (w/w%) (ball mill grinding) to 75.2 (w/w%) (high shear mixing).

### 3.7 γ-PGA yields from *Laminaria digitata* complex media at different cultivation times during the year

**FIGURE 11:**
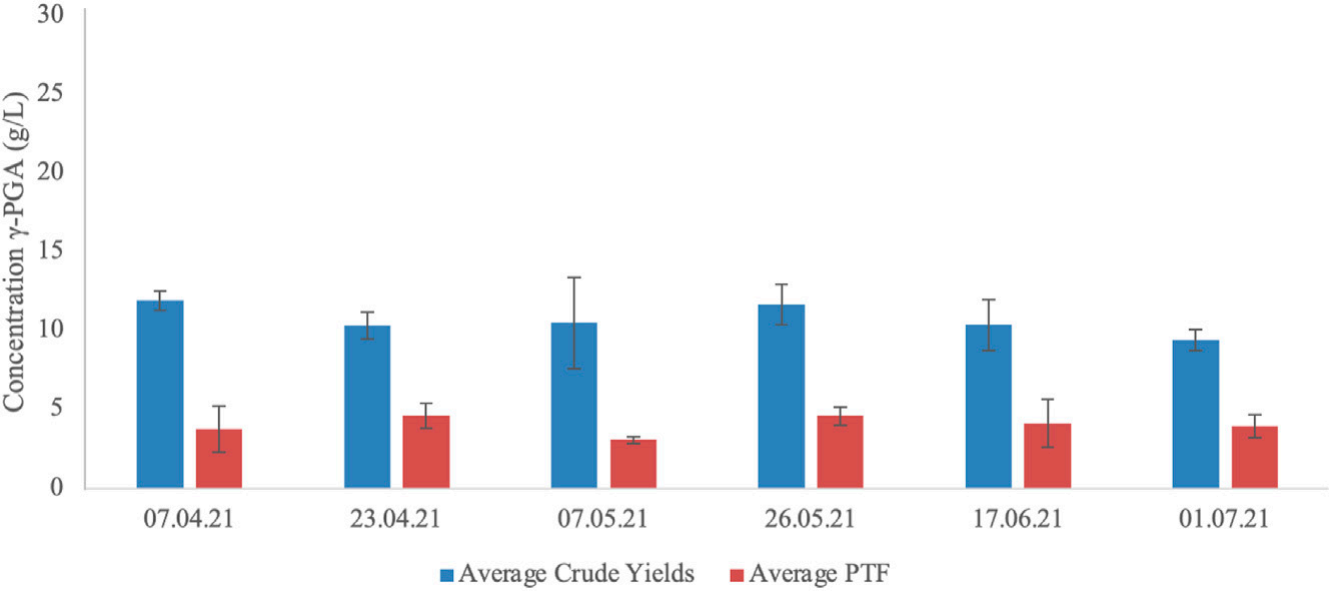
Average γ-PGA yields (raw precipitate and PTF) obtained from supplemented *Laminaria digitata* collected at different points in the year. Error bars indicate standard deviation (n = 3).

Subsequently, the effect of algal cultivation year timepoint on γ-PGA yields was assessed, the results of this investigation have been summarized within Figure 11. Similarly, to standard media cultivation, a significant difference in yields was observed between raw and purified precipitates (*p* < 0.0001). Differently, no significant difference was recorded between yearly timepoint collection for raw or purified γ-PGA yields (*p* = 0.41 and *p* = 0.46 respectively).

### 3.8 Variation in γ-PGA yields from *Alaria esculenta*, *Laminaria digitata* and *Saccharina latissima* complex media at different yearly timepoints

**FIGURE 12:**
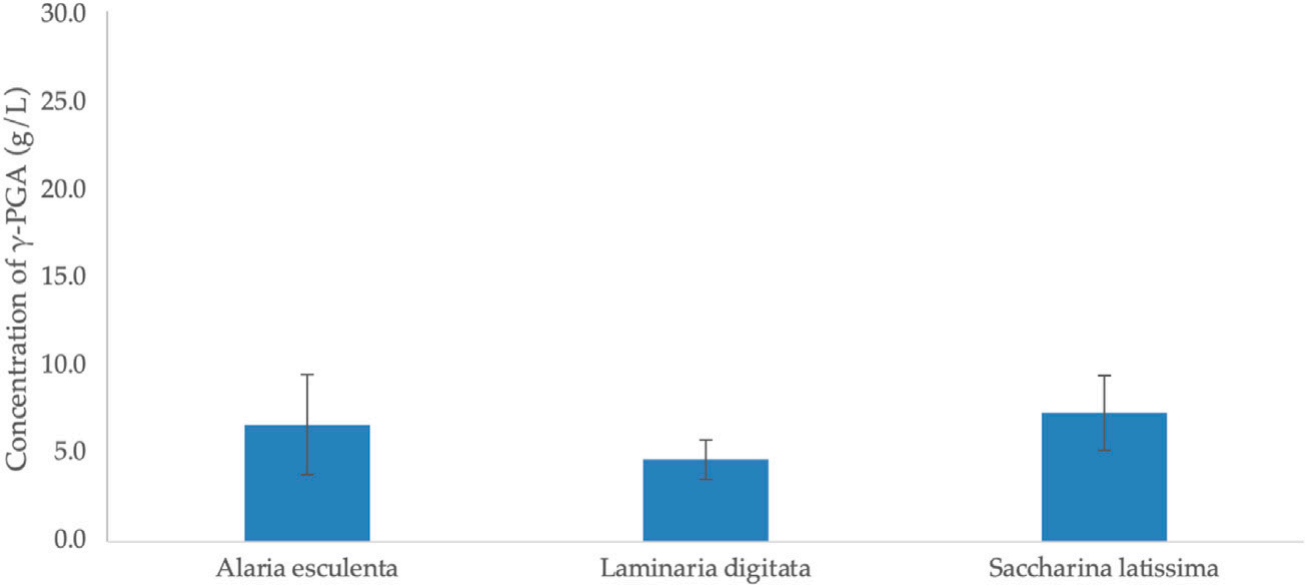
Average γ-PGA yields (PTF) obtained from supplemented *Alaria esculenta* or *Laminaria digitata* or *Saccharina latissima* cultivated in shake flasks. Error bars indicate standard deviation of six timepoints during the year.

Further, the average yields of γ-PGA from three different algal strains across the cultivation period (04.2021–07.2021) were investigated. Here media comprised of *Alaria esculenta* or *Laminaria digitata* or *Saccharina latissima* were cultivated for γ-PGA biosynthesis. The variation in γ-PGA yields from the six yearly timepoints, for each strain, is indicated by the standard deviation. All three macroalgae were cultivated in the same geographical region in the same year, which allows us to clearly analyse the extent in variation of γ-PGA yields. The results from these investigations have been summarized within Figure 12. Although variation in γ-PGA yields is evident (see standard deviation bars in Figure 12), statistical analysis suggests no significant variation in yields of γ-PGA, between the three macroalgae (*p* = 0.11). As previously displayed within Figure 10, γ-PGA yields were not found to be significantly different across the yearly timepoint.

### 3.9 Physical properties of γ-PGA produced

Although polymeric yields significantly contribute towards the overall costs of the process, one of the main aspects which determine the value of the material are its properties. To this end, the physico chemical properties of the material were analysed and summarized within Table 2.

**TABLE 2:** Physical characteristics of commercial γ-PGA and γ-PGA synthesised from GS/algal media. Samples marked with * indicate highly aggregated material; for which calculation was carried out only for the range where there was good separation.

Production method	Mn [g/mol]	Mw [g/mol]	Mw/Mn	XRD
YR spec γ-PGA	250 000	440 000	1.8	Amorphous
GS, 0 g/L NaCl PTF (250 mL)	3 320 000	3 700 000	1.1	Amorphous
GS, 25 g/L NaCl PTF (250 mL)	1 900 000	2 310 000	1.2	Semi-crystalline
GS, 50 g/L NaCl PTF (250 mL)	1 810 000	2 700 000	1.5	Crystalline
GS, 25 g/L NaCl + MnSO_4_ PTF (250 mL)	740 000	1 280 000	1.7	Crystalline
GS, 0 g/L NaCl PTF (4,000 mL)	2 840 000	3 250 000	1.1	Amorphous
GS, 25 g/L NaCl PTF (4,000 mL)	2 600 000	3 070 000	1.2	Semi-crystalline
GS, 50 g/L NaCl PTF (4,000 mL)	6 300 000 490 000	7 300 000 660 000	1.2 1.4	Semi-crystalline
GS, 25 g/L NaCl + MnSO_4_ PTF (4,000 mL)	1 200 000	1 300 000	1.1	Semi-crystalline
A.e. PTF - 07.04.21 (*)	430 000	616 000	1.4	Semi-crystalline
L.d. PTF– 07.04.21 (*)	1 760 000	2 200 000	1.3	Semi-crystalline
S.l. PTF - 07.04.21 (*)	3 400 000	4 500 000	1.3	Amorphous
A.e PTF - 07.05.21 (*)	1 400 000	2 400 000	1.7	Semi-crystalline
L.d. PTF—07.05.21 (*)	500 000	700 000	1.4	Semi-crystalline
S.l. PTF - 07.05.21 (*)	1 400 000	1 900 000	1.4	Amorphous
A.e PTF - 01.07.21 (*)	Sample fully aggregated, impossible to calculate			Semi-crystalline
L.d. PTF—01.07.21 (*)	2 400 000	4 500 000	1.8	Amorphous
S.l. PTF - 01.07.21 (*)	1 500 000	2 800 000	1.9	Amorphous
C_L.d. mechanical grinding PTF	143 000	390 000	2.7	Amorphous
C_L.d. ball mill grinding PTF	135 000	253 000	1.9	Amorphous
C_L.d. high shear mixing PTF	59 000	183 000	3.1	Amorphous

GS, Standard GS, media; PTF, Post Tangential Flow; *highly aggregated material; for which calculation was carried out only for the range where there was good separation; A. e.—*Alaria esculenta*; L.d.—*Laminaria digitata*; S.l.—*Saccharina latissima*; C_L.d.—Commercial *Laminaria digitata*.

## 4 Discussion

Although extremely versatile, γ-PGA has long been recognised as an expensive bioproduct (Nair et al., 2021). To this end, numerous attempts have been made to enhance the biosynthesis and reduce the cost of this material. Attempts have mainly focused on the substrate composition (Kedia et al., 2010; Nair et al., 2021), vessel configuration (Tang et al., 2015) and growth conditions (Shi et al., 2006). Herewith we have attempted to closely correlate macroalgal pre-treatment with γ-PGA yields and characteristics. Worldwide interest and focus towards macroalgae is well based, as these autotrophic organisms grow extremely fast, mainly require carbon dioxide and can be cause of toxic blooms. To compare the yields and properties of γ-PGA between standard media and complex supplemented algal media, yields and characteristics of γ-PGA synthesised by *Bacillus subtilis* natto on a common defined media (GS media) was established. Growth curves from Figure 3 suggest that high concentrations of NaCl (50 g/L) from standard GS media result in decreased cell viability between t = 72 and t = 96 h (*p* < 0.0001). Although cell viability was lower compared to 0 g/L NaCl, the relatively unaltered growth of the microorganism between t = 0 and t = 72 is particularly interesting as it suggests that *B. subtilis* natto can grow in osmotically challenging conditions, hinting towards energetically advantageous halophilic γ-PGA production. One of the main reasons for such a decline in cell biomass can be the increased viscosity of the media as γ-PGA is synthesised, which can significantly lower permeation capacity of oxygen, challenging enough for some of the cells to enter stationary phase (Cromwick et al., 1996; Bajaj and Singhal, 2010; Kedia et al., 2010; Ma et al., 2019; Obruca et al., 2021). This hypothesis was confirmed by 4-L fermentation studies whereby, *Bacillus subitilis* natto was cultivated on analogous 50 g/L NaCl GS media but in a fermenter with dissolved oxygen set above 40% (see Figure 4). Interestingly, other than the t = 96 h timepoint, no significant variation in growth was observed (*p* > 0.05). This suggests that adequate aeration is mostly achieved in 500 mL Erlenmeyer flasks thus allowing for comparable data to be obtained between the systems (Figure 5).

It has been previously reported in literature that supplementation of GS media with low concentrations of MnSO_4_ can significantly improve the growth of some *Bacillus* sp. (Kedia et al., 2010; Yamashiro et al., 2010). In Figure 3 we investigate such observations with *B. subtilis* natto. Although final cell concentrations of *B. subtilis* natto appears to be the same, our data suggests that 0.42 mM MnSO_4_ significantly (*p* < 0.0001) improves cellular proliferation and viability at 25 g/L NaCl concentrations compared to standard GS media (50 g/L). It has been debated that the mechanism underlying such improvement in cell viability involves the catalytic mechanism of manganese on key cellular enzymes (Leonard et al., 1958; Cromwick and Gross, 1996); with similar results also reported by Kedia et al. (2010), Leonard et al. (1958) and Cromwick and Gross (1996). Additionally, the effect of NaCl concentrations on cell counts was investigated. Here we report that, coherently to previously published literature (Birrer et al., 1994; Shimizu, et al., 2007), a decrease in NaCl concentrations is correlated to improved cellular growth compared to standard GS media.

With our set of standard media data providing coherent results when compared to what was previously reported in literature, we investigated the use of complex algal media as a suitable source of nutrients for γ-PGA production. Firstly, the pre-processing of the biomass was assessed to evaluate whether common techniques could significantly affect both the yields and the properties of γ-PGA produced. Herewith, commercial, food grade, flakes of *Laminaria digitata* dry biomass were subjected to three mechanical processes: mortar and pestle, ball mill grinding and high shear mixing. Particle size analysis suggests that, compared to initial flakes, all pre-treatment techniques reduced the particle size of the material significantly (Figure 5[A]). There were however incongruencies related to qualitative appearance of particle sizes and quantitative measured particle sizes recorded between standard commercial flakes, mortar and pestle pre-treated flakes and ball mill ground flakes (Figure 5[A]; Figure 6[V&VI]). The samples were analysed further for a total of 10 replicates to further understand why such qualitative variation was not translated quantitative into particle size measurements. Such variation in particle size can be a direct consequence of the particle size analyser limit which presents analysis hole diameter at 5 mm. The overall dimensions of irregular algal flake particles were measured with a precision digital Vernier calliper and found to range between 1.1 and 16 mm (data not shown, refer to Figure 6). Further, according to the manufacturer, upper limits of particle detection are 3.5 mm although analysis hole diameter is 5 mm; and thus, only particles with a consistent diameter smaller than 3.5 mm could be analysed.

The growth of *B. subtilis* natto at different cultivation time points was significantly different (*p* = 0.01) across the three pre-treatment techniques. Such variation in cellular density across the three groups is expected as the different techniques would incur in different particle size with variable surface area and variable extraction capacity of sugars, minerals, and other soluble components (Xu et al., 2008). However, although different across the three groups, all conditions lead to similar cell densities; between t = 72 and t = 96 h (see Figure 7). When the yields of γ-PGA were investigated across the three groups, higher surface area to volume ratios expected through ball mill grinding were shown to not be as high as those obtained through high shear mixing (Figure 5[A] and 6 [VI&VII]). Complementing the results obtained within Figure 5[A], overall higher yields of γ-PGA were obtained post tangential flow with high shear mixing compared to ball mill grinding (see Figure 10). In this respect, it is suggested that rupturing of the algal cell wall in water leads to better extraction of nutrients compared to dry algal milling. This result is particularly positive as, within an industrial context, the energy consumption of high shear mixing is significantly lower compared to those of ball mill grinding.

The chemical composition of all precipitates was investigated with the aid of FT-IR analytical techniques. Figure 8 displays very limited variation in size and location of functional groups, suggesting that the chemical composition of the compounds assessed is very similar. We appear to have analogous chemical compositions; however, it is very likely that the molar mass and crystallinity of the material is different compared to that of standard GS media.

To remove low molar mass fractions within the ethanol precipitates as well as the impurities observed within Figure 8, we employed tangential flow filtration, whereby our ethanol precipitate is passed through a 30 kDa membrane. Overall, both shake flasks and 4-L fermenters suggested significant variation in both raw yields (*p* < 0.0001 and *p* = 0.0016 respectively one way ANOVA) and Post Tangential Flow (PTF) yields (*p* = 0.0004 and *p* = 0.0018 respectively one way ANOVA). This is expected as the variation in NaCl and MnSO_4_ has been already reported as significantly affecting the yields of the material. Further, in the case of any fermentation media, it is well recognised that several low molar mass impurities can be isolated with standard ethanol precipitation, as such it is not a surprise that the yields are different between the two groups (Manocha and Margaritis, 2010).

Figure 9 suggests that for 25 g/L NaCl GS media shake flask cultivation, the highest yields of γ-PGA are obtained with MnSO_4_ (see [B]). This result is in line with what reported by Kedia et al. (2010) and previously by Ashiuchi et al. (1999). However, opposite results are observed in 4-L fermenters, wherein the 25 g/L NaCl without MnSO_4_ provided the highest yields. Such discrepancy could be induced by the lack of challenge (high availability of oxygen) present in fermenters or by the variation in extrachromosomal DNA maintenance expression described by Yamashiro et al. (2010) for which Mn^2+^ can act as an inhibitory molecule (see Figure 9[A]). Figure 9 also suggests that there is significant difference in yields (after tangential flow) between 0 and 50 g/L NaCl media in shake flasks (*p* = 0.0083) but not in 4-L fermenters (*p* = 0.99). This is particularly surprising as we would expect to have analogous results for both shake flask and 4-L fermenters; given that unvaried amounts of nutrients are present in both media. From this we infer that the only factor which might have increased γ-PGA biosynthesis at 0 g/L in shake flasks more than in Fermenters is the lower availability of oxygen, a stimulatory challenge, which is not as strong in 4-L fermenters. Across all cultivation conditions, when the material is further purified by means of tangential flow, yields obtained from shake flasks were significantly different (higher) compared to those obtained from 4-L fermenter (*p* = 0.0164). As previously mentioned, in shake flasks the lower availability of oxygen can significantly increase the overall stress on the microorganism which leads to the production of higher molar mass γ-PGA.

After having established that flask cultivation can provide a suitable representation of γ-PGA yields with standard GS media, we investigated the yields obtained from supplemented algal media. Data summarised within Figure 10 suggests that there is a significant difference in γ-PGA yields between raw and post tangential flow treatment samples of pre-treated *L. digitata* flakes (*p* = 0.0001). This follows our hypothesis as there is an incredible diversity in molar mass of components isolated within the primary ethanol precipitate, which is then removed through tangential flow filtration. As expected, the percentage of γ-PGA following tangential flow treatment (compared to raw precipitate) increased between pre-treatment methods from 37.5 (w/w%) (mortar and pestle) to 59.6 (w/w%) (ball mill grinding) to 75.2 (w/w%) (high shear mixing). Such results appear to be very promising provided that standard GS media can yield significantly lower amounts of γ-PGA (38.9 w/w% γ-PGA in shake flasks and 58.7 w/w% in 4-L fermenters) (see Figure 9). Regardless, it is very important to underline that such variation in yields after purification can be due to the difference in molar mass of the material produced compared to the actual amounts analysed after tangential flow treatment. In this respect it is possible that ball mill grinding, for example, leads to higher amounts of low chain length γ-PGA which might not be retained from tangential flow filtration. This hypothesis can be in part supported by the similar amounts of raw precipitates observed between ball mill grinding and high shear mixing (Figure 10).

Once the optimal pre-treatment method was established, macroalgal samples originating from cultivations from the Scottish Association for Marine Science research facility, were employed to assess the variation in γ-PGA yields and characteristics across macroalgal strains. When *L. digitata*, collected at different yearly timepoints was pre-treated by means of high shear mixing and then was subsequently used for γ-PGA production, there was no significant difference between yearly timepoint collection and raw or purified γ-PGA yields (*p* = 0.41 and *p* = 0.46 respectively) (Figure 11). This suggests that the variation in polysaccharides and other micro/macro molecules of *L. digitata* during the year does not significantly impact the yields of γ-PGA. Perhaps *B. subtilis* natto can utilise all the different components effectively for both metabolic activities and γ-PGA production. Although no significant variation was observed between γ-PGA yields PTF, this does not preclude a lack of variation in molar mass of γ-PGA from different yearly timepoints. This has been further explored in Table 2 and discussed subsequently.

Cumulative γ-PGA yields, from post tangential flow purification, of three different brown macroalgal strains (*A. esculenta*, *L. digitata* and *Saccharina latissima*) have been summarized within Figure 12. The standard deviation of the data displayed in Figure 12 summarises an average of the three algal strains collected across six intervals across the same year (07.04.21, 23.04.21, 07.05.21, 26.05.21, 17.06.21, 01.07.21). It has been widely reported that at increasing yearly cultivation, the biochemical composition of the three macroalgae changes significantly, however, post tangential flow filtration of γ-PGA does not appear to suggest enormous variation in yields across the three species (Schiener et al., 2014). Herewith it is suggested that *Alaria esculenta* is the one macroalgae which stimulates the bigger variation in γ-PGA yields; perhaps this follows the season variation in polysaccharide abundance, which can increase up to 30.3% ± 1.5% of the total weight between May and July as reported by Schiener et al. (2014).

Subsequently, the average γ-PGA yields of *A. esculenta* or *S. latissima* or *L. digitata* at different yearly cultivation timepoints was investigated. Differently from *L. digitata* only (Figure 11) the average of the three algae varies significantly more across the timepoints compared to *L. digitata* alone (standard deviation values Figure 11). Such high dispersity in γ-PGA values suggests that the components of the three algae, and their amounts, significantly influence the overall biosynthesis of γ-PGA. These results are logical when considering what has been discussed and reported by Schiener et al. (2014) who cultivated the same species in the same geographical region and observed that alginate formed most of the carbohydrate content in *L. digitata*, *S. latissima* and *A. esculenta* at 34.6 ± 3.1, 28.5% ± 3.9% and 37.4% ± 4.0% of the dry weight, respectively. Further, average glucan levels (a combination of laminarin and cellulose) were similar across *L. digitata*, *S. latissima* and *A. esculenta at* 18.7 ± 5.2, 19.2 ± 6.2, to 22.5% ± 7.6% of dry matter respectively (Schiener et al., 2014). In *A. esculenta* samples, the total carbon content was found to be 30.3% ± 1.5% of the total weight, which was highest between May and July; in line with the higher yields observed for *A. esculenta* compared to the other two species (Figure 12). Schiener et al. (2014) suggest that all three species saw the least amount of carbon in March; understandable as between February and May the highest amounts of nitrogen were recorded. For these algae, nitrogen content was lowest in autumn (Schiener et al., 2014). Average nitrogen-to-protein conversion factors (Jones’ factor) of 4.7 for *L. digitata,* 5.3 for *S. latissima* and 6.0 for *A. esculenta* were calculated by Schiener et al. (2014). N-conversion values are generally found to be lower than the traditional factor of 6.25, possibly due to the presence of non-protein N-containing compounds (Lourenço et al., 1998; Lourenço et al., 2004). These non-protein N-containing compounds might have a crucial role as precursors for γ-PGA biosynthesis, as could easily be converted to α-ketoglutarate or L/D-glutamate directly. Also, others have previously investigated the variation in micro and macro elements in the three species of brown seaweed. In fact, Adams et al. (2011a) reports that the highest alginate content in kelp species occur in summer months. The storage carbohydrates mannitol and laminarin in Laminariales have also been found to accumulate during summer and autumn (Rosell and Srivastava, 1984; Adams et al., 2011a) Another significant part of brown seaweed biomass is its ash content which can account for over 50% of its dry weight (Moss, 1952). The ash content of the kelp species *L. digitata* consists largely of the ions—sodium, potassium, calcium, and magnesium—with chloride and sulphate as the main counter-ions (Adams et al., 2011b).

It has been reported that γ-PGA is an exo-pseudopolypeptide that is particularly abundant when extracellular conditions are challenging. Although several groups report how different environmental conditions stimulate biosynthesis of γ-PGA, here it appears that *B. subtilis* natto is capable of synthesising high molar mass material just in the presence of copious amounts of the precursor glutamic acid. In fact, in GS, 0 g/L NaCl PTF, a 3,700 kDa material is synthesized. Our data shows that the lack in NaCl leads to the formation of amorphous material. This set of results suggests that in the GRAS organism *B. subtilis* natto, γ-PGA biosynthetic enzymatic cassette genes have a particularly strong promoter region. Surprisingly the molar mass of γ-PGA synthesised with 25 and 50 g/L of NaCl yielded materials very similar in properties. This might suggest that, in *B. subtilis* natto, the presence of NaCl in the media is an on/off switch towards the biosynthesis of γ-PGA. Although racemisation enzymes have been suggested to be altered by the present of multivalent metallic ions (Na^+^ is not considered, as monovalent) there is a difference in the crystallinity of the material (Wu et al., 2006). This result suggests that, in *B. subtilis* natto the presence of NaCl has a significant impact on the racemisation of the glutamic acid monomers. Although extensively reported in the case of MnSO_4_, modulatory effects of NaCl towards γ-PGA racemisation have not been reported previously for *B. subtilis* natto (Luo et al., 2016; Parati et al., 2022) although reported for *Bacillus megaterium* (Shimizu et al., 2007). This suggests that the behaviour of *B. subtilis* natto is somewhat similar to other *Bacillus* sp. γ-PGA producers (i.e., *licheniformis* and *megaterium*) yet it can behave quite differently. It was previously reported that unlike *Bacillus anthracis*, *B. subtilis* natto carries γ-PGA synthesising genes within its chromosome rather than a plasmid (Ashiuchi et al., 1999). Further, differently to the human pathogen *B. subtilis* 168, the expression of γ-PGA in *B. subtilis* natto is regulated by extrachromosomal DNA maintenance-based processes. Similarly, to the PGS cassette of genes, extrachromosomal DNA elements can be altered by the presence of metals. Yamashiro et al. (2011). Yamashiro et al. (2010) discusses how the production of γ-PGA can be enhanced in *B. subtilis* natto in the presence of Zn^2+^ and how it can be inhibited by the presence of Mn^2+^. Perhaps this is the reason why the overall molar mass of γ-PGA was found to be lower in a 25 g/L NaCl medium supplemented with 2.48 mM of MnSO_4._ Such behaviour is particularly interesting as it has not been previously reported for *B. subtilis* natto. Interestingly, Gross (1998) does investigate the effect of γ-PGA isolation time on the molar mass of the material and suggests that in the presence of MnSO_4_, isolation of the material at t = 140 h leads to far lower masses compared to t = 22 h. Theoretically, such a variation in molar mass should lead to proportionally higher dispersity rates of the material isolated, which is in fact what we observe in this instance. When the material is synthesised within a 4-L fermenter, the properties of 0 g/L NaCl γ-PGA are very similar to those obtained in shake flasks. Differently however, both molar masses and crystallinity of 25 and 50 g/L NaCl are significantly different compared to those obtained through shake flasks. As previously mentioned, the main factors which could affect such variation are the working volume, the stirring speeds, and the oxygen permeation.

When the three species of cultivated macroalgae, in analogous geographical region, were employed as substrate for γ-PGA production, very interesting variation in physical properties were observed: For *A. esculenta*, at increasing isolation timepoints the molar masses of γ-PGA produced increased, whereas the crystalline behaviour remained unchanged. For *L. digitata* the molar masses were very similar at the beginning and end of cultivation (April and July respectively), with a significant decrease in May. Further although similar molar masses were observed, a semi-crystalline material was produced in April whereas an amorphous material was synthesised in July. For *S. latissima* a similar decrease in molar mass was observed in May compared to April and July, however the γ-PGA produced in April had a higher molar mass compared to July and all polymers produced displayed amorphous behaviour throughout the three timepoints.

## 5 Conclusion

With an overall outlook towards achieving circular biomaterial production, we evaluated the potential use of marine macroalgae to produce versatile biopolymer of poly-γ-glutamic acid. Although it was previously reported that, the supplementation of *Ulva* sp. macroalgae with 7 g/L glutamic acid could lead to the biosynthesis of γ-PGA by *Bacillus* sp., our investigation extended significantly beyond that, and aimed to assess the optimal mechanical pre-treatment method as well as yearly time harvest for three commonly cultivated macroalgae in the Northern part of Europe: namely, *A. esculenta*, *S. latissima* and *L. digitata*. Firstly, we concluded that out of three common pre-treatment methods, high shear mixing was the most suitable for γ-PGA synthesis. We then, following coherent data for both Erlenmeyer 500 mL shake flasks and 4-L benchtop fermenters, evaluated γ-PGA yields of the three macroalgae. Our data suggests that both *L. digitata* and *S. latissima* were able to support the synthesis of high molar mass amorphous γ-PGA (4,500 kDa) when isolated at opposite cultivation timepoints - in July and April respectively. We also conclude that when collected in May, overall, the three macroalgae lead to the highest yields of γ-PGA. From standard GS media we concluded that *B. subtilis* natto can grow in osmotically challenging conditions, hinting towards energetically advantageous, non-sterile, halophilic γ-PGA production. In Erlenmeyer flaks, 25 g/L NaCl GS media supplemented with 2.48 mM of MnSO_4_ leads to the highest yields of γ-PGA. In 4-L fermenter, the presence of Mn^2+^ ions might have inhibitory activity towards high γ-PGA biosynthesis in line with what previously observed. A consolidated bioprocess developed could be further expanded to target future applications under the context of the bio-economy era.

## Data Availability

The original contributions presented in the study are included in the article/Supplementary materials, further inquiries can be directed to the corresponding authors.

## References

[B1] Adams J. M. M. Ross A. B. Anastasakis K. Hodgson E. M. Gallagher J. A. Jones J. M. (2011a). Seasonal variation in the chemical composition of the bioenergy feedstock Laminaria digitata for thermochemical conversion. Bioresour. Technol. 102 (1), 226–234. 10.1016/j.biortech.2010.06.152 20685112

[B2] Adams J. M. M. Toop T. A. Donnison I. S. Gallagher J. A. (2011b). Seasonal variation in Laminaria digitata and its impact on biochemical conversion routes to biofuels. Bioresour. Technol. 102 (21), 9976–9984. 10.1016/j.biortech.2011.08.032 21900006

[B5] Ashiuchi M. Soda K. Misono H. (1999). A poly-γ-glutamate synthetic system of Bacillus subtilis IFO 3336: Gene cloning and biochemical analysis of poly-γ-glutamate produced by *Escherichia coli* clone cells. Biochem. Biophysical Res. Commun. 263 (1), 6–12. 10.1006/bbrc.1999.1298 10486244

[B50] Bai N. He Y. Zhang H. Zheng X. Zeng R. Li Y. (2022). γ-Polyglutamic acid production, biocontrol, and stress tolerance: multifunction of bacillus subtilis A-5 and the complete Genome Analysis. Int. J. Environ. Res. Public Health 19 (13), 7630. 10.3390/ijerph19137630 35805288 PMC9265942

[B6] Bajaj I. B. Singhal R. S. (2010). Effect of aeration and agitation on synthesis of poly (γ-glutamic acid) in batch cultures of Bacillus licheniformis NCIM 2324. Biotechnol. Bioprocess Eng. 15 (4), 635–640. 10.1007/s12257-009-0059-2

[B8] Birrer G. A. Cromwick A.-M. Gross R. A. (1994). γ-Poly(glutamic acid) formation by Bacillus licheniformis 9945a: Physiological and biochemical studies. Int. J. Biol. Macromol. 16 (5), 265–275. 10.1016/0141-8130(94)90032-9 7534473

[B11] Cromwick A. Birrer G. Gross R. (1996). Effects of pH and aeration on gamma-poly(glutamic acid) formation by Bacillus licheniformis in controlled batch fermentor cultures. Biotechnol. Bioeng. 50. 10.1002/(SICI)1097-0290(19960420)50:2<222:AID-BIT10>3.0.CO;2-P 18626940

[B51] Deol R. Louis A. Glazer H. L. Hosseinion W. Bagley A. Chandrangsu P. (2022). Poly-Gamma-Glutamic acid secretion protects bacillus subtilis from zinc and copper intoxication. Microbiol. Spectr. 10 (2). 10.1128/spectrum.01329-21 PMC9045300 35311566

[B12] Dunn J. B. Biddy M. Jones S. Cai H. Benavides P. T. Markham J. (2017). Environmental, economic, and scalability considerations and trends of selected fuel economy-enhancing biomass-derived blendstocks. ACS Sustain. Chem. Eng. 6 (1), 561–569. 10.1021/acssuschemeng.7b02871

[B52] Inbaraj B. S. Chien J. T. Ho G. H. Yang J. Chen B. H. (2006). Equilibrium and kinetic studies on sorption of basic dyes by a natural biopolymer poly(γ-glutamic acid). Biochem. Eng. J. 31 (3), 204–215. 10.1016/j.bej.2006.08.001

[B17] Kedia G. Hill D. Hill R. Radecka I. (2010). Production of poly-γ-glutamic acid by Bacillus subtilis and;Bacillus licheniformi with different growth media. J. Nanosci. Nanotechnol. 10 (9), 5926–5934. 10.1166/jnn.2010.2614 21133130

[B18] Khalil I. Burns A. Radecka I. Kowalczuk M. Khalaf T. Adamus G. (2017). Bacterial-derived polymer poly-y-glutamic acid (y-PGA)-Based micro/nanoparticles as a delivery system for antimicrobials and other biomedical applications. Int. J. Mol. Sci. 18 (2), 313. 10.3390/ijms18020313 28157175 PMC5343849

[B19] Kim J. Lee J. M. Jang W. J. Park H. D. Kim Y. Kim C. (2019). Efficient production of poly γ-d -glutamic acid from the bloom-forming green macroalgae, *Ulva* sp., by *Bacillus* sp. SJ-10. SJ-10. *Biotechnol. Bioeng.* 116 (7), 1594–1603. 10.1002/bit.26966 30883680

[B22] Leonard C. G. Housewright R. D. Thorne C. B. (1958). Effects of some metallic ions on glutamyl polypeptide synthesis by bacillus subtilis. J. Bacteriol. 76 (5), 499–503. 10.1128/jb.76.5.499-503.1958 13598708 PMC290228

[B23] Lourenço S. O. Barbarino E. Lavín P. L. Lanfer Marquez U. M. Aidar E. (2004). Distribution of intracellular nitrogen in marine microalgae: Calculation of new nitrogen-to-protein conversion factors. Eur. J. Phycol. 39 (1), 17–32. 10.1080/0967026032000157156

[B24] Lourenço S. O. Barbarino E. Marquez U. M. L. Aidar E. (1998). Distribution of intracellular nitrogen in marine microalgae: Basis for the calculation of specific nitrogen-to-protein conversion factors. J. Phycol. 34 (5), 798–811. 10.1046/j.1529-8817.1998.340798.x

[B25] Luo Z. Guo Y. Liu J. Qiu H. Zhao M. Zou W. (2016). Microbial synthesis of poly-γ-glutamic acid: Current progress, challenges, and future perspectives. Biotechnol. Biofuels 9 (1), 134. 10.1186/s13068-016-0537-7 27366207 PMC4928254

[B26] Ma X. Zhu S. Luo M. Hu X. Peng C. Huang H. (2019). Intracellular response of Bacillus natto in response to different oxygen supply and its influence on menaquinone-7 biosynthesis. Bioprocess Biosyst. Eng. 42 (5), 817–827. 10.1007/s00449-019-02085-x 30758672

[B27] Manocha B. Margaritis A. (2010). A novel Method for the selective recovery and purification of γ-polyglutamic acid fromBacillus licheniformisfermentation broth. Biotechnol. Prog. 26 (3), 734–742. 10.1002/btpr.370 20063385

[B53] Miles A. A. Misra S. S. Irwin J. O. (1938). The estimation of the bactericidal power of the blood. Epidemiol. Infect. 38 (6), 732–749. 10.1017/s002217240001158x PMC2199673 20475467

[B28] Moss B. (1952). Variations in chemical composition during the development of Himanthalia elongata (L.) S. F. Gray. J. Mar. Biol. Assoc. U. K. 31 (1), 29–34. 10.1017/s0025315400003659

[B29] Nair P. G. Joseph E. Killi N. Konchada S. Nisal A. Gundloori R. V. N. (2021). One-pot bioconversion of tomato waste into poly-gamma-glutamic acid (γ-PGA) biopolymer by a novel biocatalyst. ACS Sustain. Chem. Eng. 9 (43), 14330–14334. 10.1021/acssuschemeng.1c05362

[B30] Nair P. Navale G. R. Dharne M. S. (2021a). poly-gamma-glutamic acid biopolymer: A sleeping giant with diverse applications and unique opportunities for commercialization. Biomass Convers. Biorefinery 13, 4555–4573. 10.1007/s13399-021-01467-0 PMC8016157 33824848

[B31] Obruca S. Sedlacek P. Koller M. (2021). The underexplored role of diverse stress factors in microbial biopolymer synthesis. Bioresour. Technol. 326, 124767. 10.1016/j.biortech.2021.124767 33540213

[B54] Ogawa Y. Yamaguchi F. Yuasa K. Tahara Y. (1997). Efficient production of γ-Polyglutamic acid by Bacillus subtilis(natto) in jar fermenters. Biosci. Biotechnol. Biochem. 61 (10), 1684–1687. 10.1271/bbb.61.1684 27393164

[B32] Ogunleye A. Bhat A. Irorere V. U. Hill D. Williams C. Radecka I. (2015). Poly-γ-glutamic acid: Production, properties and applications. Microbiology 161 (1), 1–17. 10.1099/mic.0.081448-0 25288645

[B34] Parati M. Clarke L. Anderson P. Hill R. Khalil I. Tchuenbou-Magaia F. (2022a). Microbial poly-γ-glutamic acid (γ-PGA) as an effective tooth enamel protectant. Polymers 14 (14), 2937. 10.3390/polym14142937 35890712 PMC9317725

[B35] Parati M. Khalil I. Tchuenbou-Magaia F. Adamus G. Mendrek B. Hill R. (2022b). Building a circular economy around poly(D/L-γ-glutamic acid)- a smart microbial biopolymer. Biotechnol. Adv. 61, 108049. 10.1016/j.biotechadv.2022.108049 36243207

[B36] Pardilhó S. Cotas J. Pereira L. Oliveira M. B. Dias J. M. (2022). Marine macroalgae in a circular economy context: A comprehensive analysis focused on residual biomass. Biotechnol. Adv. 60, 107987. 10.1016/j.biotechadv.2022.107987 35605758

[B37] Rosell K.-G. Srivastava L. M. (1984). Seasonal variation in the chemical constituents of the Brown algae Macrocystis integrifolia and Nereocystis luetkeana. Can. J. Bot. 62 (11), 2229–2236. 10.1139/b84-303

[B38] Schiener P. Black K. D. Stanley M. S. Green D. H. (2014). The seasonal variation in the chemical composition of the kelp species Laminaria digitata, Laminaria hyperborea, Saccharina latissima and Alaria esculenta. J. Appl. Phycol. 27 (1), 363–373. 10.1007/s10811-014-0327-1

[B39] Shi F. Xu Z. Cen P. (2006). Efficient production of poly-γ-glutamic acid by Bacillus subtilis ZJU-7. Appl. Biochem. Biotechnol. 133 (3), 271–282. 10.1385/abab:133:3:271 16720907

[B40] Shimizu K. Nakamura H. Ashiuchi M. (2007). Salt-inducible bionylon polymer from Bacillus megaterium. Appl. Environ. Microbiol. 73 (7), 2378–2379. 10.1128/AEM.02686-06 17293523 PMC1855635

[B42] Tang B. Zhang D. Li S. Xu Z. Feng X. Xu H. (2015). Enhanced poly(γ-glutamic acid) production by H2O2-induced reactive oxygen species in the fermentation ofBacillus subtilisNX-2. Biotechnol. Appl. Biochem. 63 (5), 625–632. 10.1002/bab.1416 26202728

[B43] Wu Q. Xu H. Xu L. Ouyang P. (2006). Biosynthesis of poly(γ-glutamic acid) in Bacillus subtilis NX-2: Regulation of stereochemical composition of poly(γ-glutamic acid). Process Biochem. 41 (7), 1650–1655. 10.1016/j.procbio.2006.03.034

[B44] Xu P. Ding Z.-Y. Qian Z. Zhao C.-X. Zhang K.-C. (2008). Improved production of mycelial biomass and ganoderic acid by submerged culture of Ganoderma lucidum SB97 using complex media. Enzyme Microb. Technol. 42 (4), 325–331. 10.1016/j.enzmictec.2007.10.016

[B45] Yamashiro D. Minouchi Y. Ashiuchi M. (2011). Moonlighting role of a poly-γ-glutamate synthetase component from Bacillus subtilis: Insight into novel extrachromosomal DNA maintenance. Appl. Environ. Microbiol. 77 (8), 2796–2798. 10.1128/aem.02649-10 21357437 PMC3126358

[B46] Yamashiro D. Yoshioka M. Ashiuchi M. (2010). Bacillus subtilis pgsE (Formerly ywtC) stimulates poly-γ-glutamate production in the presence of zinc. Biotechnol. Bioeng. 108 (1), 226–230. 10.1002/bit.22913 20812257

[B47] Yang L. Wang X.-C. Dai M. Chen B. Qiao Y. Deng H. (2021). Shifting from fossil-based economy to bio-based economy: Status quo, challenges, and prospects. Energy 228, 120533. 10.1016/j.energy.2021.120533

[B55] Ye H. Jin L. Hu R. Yi Z. Li J. Wu Y. (2006). Poly(gamma,L-glutamic acid)-cisplatin conjugate effectively inhibits human breast tumor xenografted in nude mice. Biomater. 27 (35), 5958–5965. 10.1016/j.biomaterials.2006.08.016 16949149

[B48] Zhang C. Wu D. Ren H. (2019). Economical production of agricultural γ-polyglutamic acid using industrial wastes by Bacillus subtilis. Biochem. Eng. J. 146, 117–123. 10.1016/j.bej.2019.03.013

[B49] Zhang L. Zhu X. Wu S. Chen Y. Tan S. Liu Y. (2018). Fabrication and evaluation of a &gamma;-PGA-based self-assembly transferrin receptor-targeting anticancer drug carrier. Int. J. Nanomedicine 13, 7873–7889. 10.2147/ijn.s181121 30538465 PMC6255109

